# Antitumor activity and mechanisms of action of total glycosides from aerial part of *Cimicifuga dahurica *targeted against hepatoma

**DOI:** 10.1186/1471-2407-7-237

**Published:** 2007-12-31

**Authors:** Ze Tian, Jianyong Si, Qi Chang, Liang Zhou, Shilin Chen, Peigen Xiao, Erxi Wu

**Affiliations:** 1 Children's Hospital Informatics Program at Harvard-MIT Division of Health Sciences and Technology, Children's Hospital Boston, Harvard Medical School, Boston, Massachusetts 02115, USA; 2 Institute of Medicinal Plant Development, Chinese Academy of Medical Sciences, Peking Union Medical College, Beijing 100094, China

## Abstract

**Background:**

Medicinal plant is a main source of cancer drug development. Some of the cycloartane triterpenoids isolated from the aerial part of *Cimicifuga dahurica *showed cytotoxicity in several cancer cell lines. It is of great interest to examine the antiproliferative activity and mechanisms of total triterpenoid glycosides of *C. dahurica *and therefore might eventually be useful in the prevention or treatment of Hepatoma.

**Methods:**

The total glycosides from the aerial part (TGA) was extracted and its cytotoxicity was evaluated in HepG2 cells and primary cultured normal mouse hepatocytes by an MTT assay. Morphology observation, Annexin V-FITC/PI staining, cell cycle analysis and western blot were used to further elucidate the cytotoxic mechanism of TGA. Implanted mouse H_22 _hepatoma model was used to demonstrate the tumor growth inhibitory activity of TGA *in vivo*.

**Results:**

The IC_50 _values of TGA in HepG2 and primary cultured normal mouse hepatocytes were 21 and 105 μg/ml, respectively. TGA induced G_0_/G_1 _cell cycle arrest at lower concentration (25 μg/ml), and triggered G_2_/M arrest and apoptosis at higher concentrations (50 and 100 μg/ml respectively). An increase in the ratio of Bax/Bcl-2 was implicated in TGA-induced apoptosis. In addition, TGA inhibited the growth of the implanted mouse H_22 _tumor in a dose-dependent manner.

**Conclusion:**

TGA may potentially find use as a new therapy for the treatment of hepatoma.

## Background

Hepatocellular carcinoma (HCC) is the fifth most common tumor worldwide, and the incidence of HCC has been rising over the past few decades in some areas such as Europe, USA and far eastern Asian countries [1]. Despite advances in diagnosis and standard therapies such as surgery, radiation, and chemotherapy, HCC remains a formidable challenge for clinical therapy [2-5]. In the search for new cancer therapeutics with low toxicity, traditional Chinese medicines are promising candidates.

The dried rhizomes of *Cimicifuga dahurica *(Turcz) Maxim (Ranunculaceae) have been used as cooling, detoxification, antipyretic and analgesic agents for the treatment of some types of headaches and toothaches in Chinese folk medicine and were included in the Chinese Pharmacopoeia [6]. The rhizomes are traditionally the portion of the plant used for medicinal purposes in *Cimicifuga *species, however the aerial part of the plant is usually discarded. Previous phytochemical studies demonstrated that both the rhizomes and the aerial part of the species are rich in cycloartane triterpenoids [7-10]. Some biological activities of total glycosides of rhizomes of *C. dahurica *(TGR) have been investigated by earlier studies of our group. It was reported that TGR could reduce the production of Simian Immunodeficiency Virus (SIV) by inhibition of PHA stimulated ^3^H-TdR transportation in lymph cells as well as suppression of the Sister Chromatid Exchange frequency induced by mitomycin C in human peripheral lymphocytes [11,12]. Nevertheless, there are still few reports on the bioactivity of the aerial part of *C. dahurica*. Our recent study has demonstrated cytotoxicity of TGA and three cycloartanes 23, 24 and 25-O-acetylcimigenol-3-O-*β*-D-xylopyranoside isolated from the aerial part of *C*.*dahurica *against several cancerous cell lines. These three compounds showed similar effects and induced apoptosis and G_2_/M cell cycle arrest in hepatoma HepG2 and leukemia HL-60 cell lines. Down regulated expression of cdc2 and COX-2 contributed to the apoptosis and cell cycle arrest in HepG2 cells [13]. However, the cytotoxic mechanism and *in vivo *anti-tumor activity of TGA is still unknown.

In the current study, we investigated the anti-tumor activity and the underlying mechanism of TGA both *in vitro *and *in vivo*. Our findings show the novel anticancer activity of TGA and this may provide a new approach to the hepatoma therapy.

## Methods

### Extraction of triterpene components from aerial part of *C. dahurica*

The aerial part of *Cimicifuga dahurica *(Turcz) Maxim (synonyms: Actinospora dahurica Turczaninow ex Fischer & C. A. Meyer, Index Sem. Hort. Petrop. 1: 21. 1835; Actaea dahurica (Turczaninow ex Fischer & C. A. Meyer) Turczaninow ex Fischer & C. A. Meyer) was collected in Maojingba, Kalaqin Qi, Inner Mongolia Autonomous Region, China, in September 1999, and was identified by Prof. Ruile Pan of the Institute of Medicinal Plant Development, Chinese Academy of Medical Sciences and Peking Union Medical College. A voucher specimen has been deposited in the Herbarium of the Institute (XA99-09). The powdered aerial part of the plant (14.5 kg) was extracted exhaustively with 10 folds volume of 80% ethanol under refluxing for three times, one hour each time. Following combination and filtering, the solvent was evaporated under vacuum to obtain the crude extract (2.0 kg). Then the crude extract was mixed with siliceous earth and eluted with ethyl acetate. Removal of the solvent in vacuo, the TGA was (210 g) obtained.

### Determination of total content of triterpenes

Twenty nine triterpene glycosides (Table 1) including 25-anhydrocimigenol-3-O-β-D-xylopyranoside, 23-O-acetylcimigenol-3-O-β-D-xylopyranoside, 24-O-acetylcimigenol-3-O-β-D-xylopyranoside, and 25-O-acetylcimigenol-3-O-β-D-xylopyranoside, cimigenol xylopyranoside together with ferulic acid and isoferulic acid were isolated from TGA. The total triterpene glycosides content in TGA was estimated by a colorimetric method as we described previously [14], with slight modifications. A 50-μl aliquot of TGA methanol solution (1.50 mg/ml) was diluted with 1 ml water and then applied to a Waters Oasis HLB cartridge, which was preconditioned by rinsing with 1 ml methanol and followed by 1 ml water. The cartridge was washed with 2 ml water to remove carbohydrate compounds for interference, and then the triterpenes were eluted with 2 ml methanol from the cartridge to a clean glass tube. After drying by a gentle stream of nitrogen, a 0.2-ml aliquot of 5% vanillin acetic acid (w/v) and a 0.8-ml of aliquot of perchloric acid were added to the residue in the tube. Then the tube was kept in a 80°C water bath for 15 min. After cooling with water, the absorbance of the mixture was determined at 544 nm. The assay was conducted in triplicates. The total triterpene glycosides content was 72.67% ± 2.03 expressed as cimigenol xylopyranoside equivalents. Usually the total triterpene glycosides content in *C. racemosa *is calculated as 27-deoxyactein equivalent [15]; However since 27-deoxyactein was not isolated from TGA, cimigenol xylopyranoside, one of the main components in TGA and many other *Cimicifuga *plants was used as a standard.

**Table 1:** Triterpene constituents from *C. dahurica *Thurez Maxim

1	Cimilactone A [12β-acetoxy-3β-β-D-xylopyrano-syloxy-24, 25, 26, 27-tetranor-9,19-cyclolanost-16, 23 -lactone]
2	Cimilactone B [12β-acetoxy-3β-β-D-xylopyranosyloxy-24, 25, 26, 27-tetranor-9,19-cyclolanost-7-ene – 16, 23-lactone]
3	Cimidahuside C [12β-acetoxy- 15-oxo-shengmanol-3-O-β-D-xylopyranoside]
4	Cimidahuside D [12β-acetoxy- 15-oxo-7, 8-didehydroshengmanol- 3-O-β-D-xylopyranoside]
5	Cimidahuside E [(20R, 24R)-24, 25-epoxy-3β-(β-D-xylopyranosyloxy)-9,19-cyclolanost-7-ene-16, 23-dione]
6	Cimidahuside F [(20R, 24R)-24, 25-epoxy-15a-hydroxy-3β-(β-D-xylopyranosyloxy)-9,19-cyclolanost-7-ene-16,23-dione]
7	Cimidahuside G [(23R,24S)-15- oxo-16-enol-9,19-cyclolanostane-3-O-β-D-xylopyranoside]
8	Cimidahuside H [(23R,24S)-15- oxo-16-enol-9, 19-cyclolanostane -7-ene-3-O-β-D-xylopyranoside]
9	Cimidahuside I [(23R, 24S)- 12β-acetoxy-15-oxo-16-enol-9,19-cyclolanostane-3-O-β-D-xylopyranoside]
10	Cimidahuside J [(23R,24S)- 12β-acetoxy-15-oxo-16-enol-9, 19-cyclolanostane-7-ene-3-O-β-D-xylopyranosid e]
14	(20R, 24R)-11β,24,25-trihydroxy -3-β-(β-D-xylopyranosyloxy)- 9,19-cyclolanost-7-ene-16,23- dione
15	25-anhydrocimigenol-3-O-β-D-xylopyranoside
16	24-epi-7,8-didehydrocimigenol -3-O-β-D-xylopyranoside
17	cimigenol-3-O-β-D- xylopyranoside
18	7,8-didehydrocimigenol-3-O-β-D-xylopyranoside
19	25-O-methylcimigenol-3-O-β-D- xylopyranoside
20	15a-hydroxycimicidol-3-O-β-D-xylopyranoside
21	7β-hydroxycimigenol-3-O-β-D-xylopyranoside
22	12β-hydroxycimigenol-3-O-β-D-xylopyranoside
23	24-O-acetyl-7, 8-didehydrocimigenol-3-O-β-D-xylopyranoside
24	24-O-acetylcimigenol-3-O-β-D-xylopyranoside
25	25-O-methyl-24-O-acetylcimigenol-3-O-β-D-xylopyranoside
26	12β-O-acetylcimiaceroside A
27	12β-O-acetylcimiaceroside B
28	cimiaceroside A
29	cimiaceroside B

### Cell culture and drug treatment

HepG2 (ATCC, Rockville, MD) cells were maintained in RPMI 1640 containing 10% FBS (Gibco, BRL, Carlsbad, CA), 2 mg/ml sodium bicarbonate, 100 μg/ml penicillin sodium salt and 100 μg/ml streptomycin sulfate. Cells were grown to 70% confluence, trypsinized with 0.25% trypsin-2 mM EDTA, and plated in 96 well plates. Mouse hepatocytes were isolated from normal CD-1 (ICR) mice (Beijing Vital Laboratory Animal Technology, Beijing, China) with enzymatic perfusion technique as we described previously [13]. The viability of the mouse hepatocytes, tested with Trypan blue was about 80%. In all experiments, cells were grown in RPMI-1640 medium with 10% FBS for 24 h prior to treatment.

TGA was dissolved in DMSO at a concentration of 250 mg/ml, then diluted in tissue culture medium and filtered before use. The final concentration of DMSO (0.1%) did not affect the cell viability.

### Cytotoxicity assay

1.5 × 10^4 ^HepG2 cells and 8 × 10^3 ^mouse hepatocytes were seeded in 96 well plates and treated with TGA or vehicle (0.1% DMSO) at various concentrations and incubated for 48 h, followed by MTT (3- [4, 5-dimethylthiazol-2-yl]-2, 5-diphenyltetrazolium bromide) assay [16]. Briefly, IC_50 _of the TGA in HepG2 cells and normal mouse hepatocytes were derived from the dose-response curves.

### Morphology observation in HepG2 cells

AO/EB (acridine orange/ethidium bromide) fluoresce staining method was used to observe the apoptosis morphological changes [17]. Briefly, HepG2 cells were cultured in 3.5 cm dishes and treated with TGA at concentration of 50 μg/ml for 0, 12, 24 and 48 h respectively. After treatment, all the cultures were incubated at 37°C, 5% CO_2 _for the indicated time. Photographs were taken under an inverted Leica fluorescence 40 × 10 microscope after staining.

### Annexin V-FITC/PI assay

Apoptosis was quantified by detecting surface exposure of phosphatidylserine in apoptotic cells using Annexin V-FITC/PI (propidium iodide) apoptosis detection kit (BD Biosciences Clontech). Cells were seeded in 3.5 cm dishes in 1 ml medium and incubated with TGA at the dose of 25, 50 and 100 μg/ml for 24 h, respectively. The adherent and floating cells were combined and treated according to the manufacturer's instruction and measured with FITC/PI staining using flow cytometry (Becton Dickinson, San Jose, CA). Apoptotic cells (annexin V^+^PI^-^) were differentiated from necrotic cells (annexin V^+^PI^+^, including apoptotic cells at late stage).

### Cell cycle analysis

HepG2 cells were treated with TGA at different concentrations (25, 50 and 100 μg/ml for 48 h) and time points (at 50 μg/ml for 12, 24 and 48 h). Then cells were collected and fixed in 70% cold ethanol (-20°C) overnight. After washing twice with PBS, cells were resuspended in PBS. RNase A (0.5 mg/ml) and PI (2.5 μg/ml) were added to the fixed cells for 30 min. The DNA content of cells was then analyzed with a FACSCalibur instrument (Becton Dickinson, San Jose, CA).

### Western blotting

After treatments, cells were washed three times with ice-cold PBS and lysed with lysis buffer (50 mM Tris-HCl, pH 7.4, 10 mM EDTA, 1% Triton X-100, 26% urea, and 1 tablet/10 ml protease inhibitor cocktail tablets). Sticky DNA was removed from lysates with a sterile toothpick. The protein concentration of the supernatant was determined by the Bradford method. The lysates were subjected to electrophoresis on a 10 % SDS-polyacrylamide gel and then transferred to a nitrocellulose membrane [18]. The nitrocellulose membrane was then incubated with mouse monoclonal anti-Bcl-2 and anti-Bax antibody (Santa Cruz Biotechnology, Santa Cruz, CA; sc-509 and sc-7480). Mouse monoclonal β-actin (Lab Vision, Fremont, CA) was used as an internal control. Secondary antibody to IgG conjugated to horseradish peroxidase was used. The blots were probed with the ECL Western blot detection system according to the manufacturer's instructions. The ratio of Bax/Bcl-2 was analyzed by pImage tool.

### Antitumor evaluation on implanted mouse H_22 _cells

Male CD-1 (ICR) mice (Beijing Vital Laboratory Animal Technology, Beijing, China), weighing 20–22 g, were used for implantation of hepatoma H_22 _cells (s.c.), which was maintained by weekly (i.p.) passage in CD-1 (ICR) mice. Ascites (0.2 ml of 1:6 dilution) from tumor-bearing mice 7 days after tumor inoculation were implanted (s.c.) into the armpit region of mice. Ten mice each were treated with either TGA (200,100 and 50 mg/kg b.w., i.g.) or vehicle, once a day for 10 days, 24 h after tumor inoculation. Cyclophosphamide (15 mg/kg b.w., i.p.) was used as a positive control. The tumor inhibition rate (TIR %) was calculated as we described previously [19]. All the animal procedures were conducted in compliance with the National Institutes of Health Guide for the Care and Use of Laboratory Animals.

### Statistics

One-way ANOVA was used and followed by Dunnett's test. p < 0.05 was considered significant.

## Results

### Cytotoxic activity

**Figure 1:**
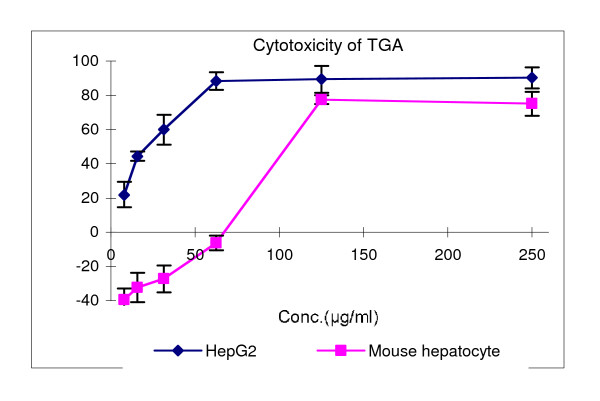
**Cytotoxic activity of TGA in HepG2 cells and normal mouse hepatocytes**. The cells were treated with vehicle or TGA from 7.8125 μg/ml to 250 μg/ml. Figure shown represents one of three independent experiments.

The cytotoxicity of TGA was evaluated by an MTT assay and the IC_50 _values were derived from the dose-response curves (Fig 1). A 48 h exposure to TGA decreased the proliferation of HepG2 cells in a concentration-dependent manner with an IC_50 _at 21 μg/ml; however, the effect on normal mouse hepatocytes showed bi-directional property: the proliferation was inhibited at higher concentrations, but promoted at lower concentrations, with an IC_50 _at 105 μg/ml. This indicates that TGA may possess relative selective cytotoxicity to hepatoma cancer cells.

### Induction of apoptosis in HepG2 cells by TGA

**Figure 2:**
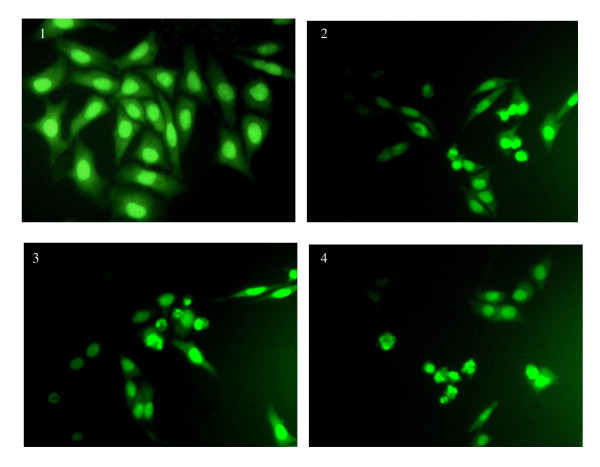
**Morphological changes of HepG2 cell line in response to TGA at 50 μg/ml for different time points**. 1–4, HepG2-cells treated with TGA at 50 μg/ml for 0, 12, 24 and 48 h, respectively.

Individual apoptosis in the cell population in HepG2 cells treated by TGA was studied by fluorescence staining method. Morphological alteration, such as chromatin aggregation, nuclear and cytoplasmic condensation, and partition of cytoplasm and nucleus into membrane-bound vesicles (apoptotic bodies) were observed in HepG2 cells treated by TGA at 50 μg/ml for 12, 24 and 48 h (Fig 2).

**Figure 3:**
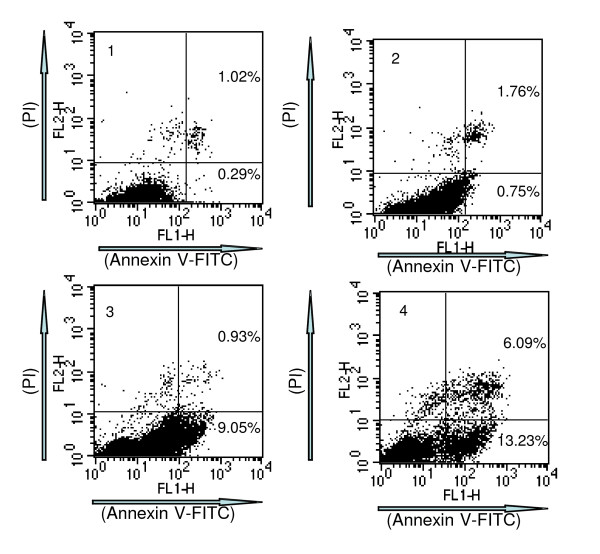
**Flow cytometric analysis of HepG2 cells treated by TGA for 24 h**. 1, Control; 2–4, HepG2 cells treatment with 25, 50 and 100 μg/ml TGA for 24 h.

To further confirm that TGA induces HepG2 cell apoptosis, cells were stained with Annexin V-FITC and PI, and then subsequently analyzed by flow cytometry. This assay is based on the translocation of phosphatidylserine from the inner leaflet of the plasma membrane to the cell surface in the early apoptotic cells. HepG2 cells were treated with TGA at 25, 50 and 100 μg/ml for 24 h. The dual parameter fluorescent dot plots showed the viable cell population in the lower left quadrant (annexin V^- ^PI^-^), the cells at the early apoptosis are in the lower right quadrant (annexin V^+^PI^-^), and the ones at the late apoptosis are in the upper right quadrant (annexin V^+^I^+^). As indicated in Fig. 3, in untreated cells, 0.29% of cells were Annexin V-positive/PI-negative, whereas 1.02% of cells were Annexin V/PI double positive. After treatment with TGA at 25, 50 and 100 μg/ml for 24 h, the corresponding quantities were 0.75 and 1.76%; 9.05 and 0.93%; 13.23 and 6.09% respectively.

### Effect of TGA on cell cycle distribution in HepG2 cells

**Figure 4:**
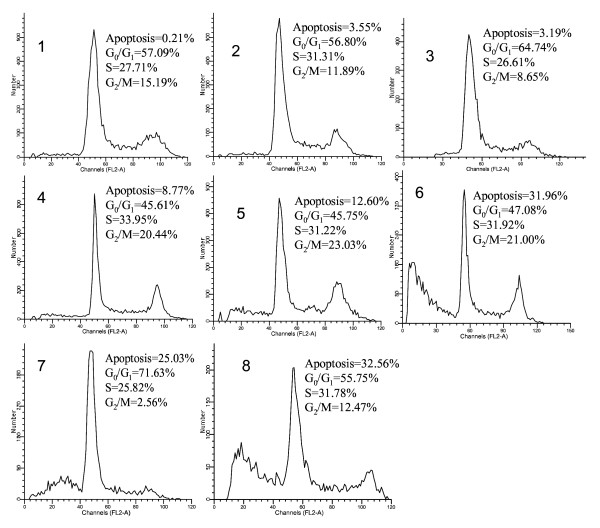
**Cell cycle distributions of HepG2 cells treated with TGA at different times and dosages**. Cells were stained with PI and analyzed by flow cytometry. 1–3, HepG2 control for 12, 24 and 48 h respectively; 4–6, HepG2 cells treated with TGA at 50 μg/ml for 12, 24 and 48 h respectively. 7–8, HepG2 cells treated with TGA at 25 and 100 μg/ml for 48 h. Figures shown are one of three representative experiments.

The ability of a substance to affect specific phases of the cell cycle may provide clues to its mechanism of action. To determine the effects of TGA on the cell cycle, HepG2 cells were treated with TGA at different concentrations (25, 50 and 100 μg/ml) and time points (at 50 μg/ml for 12, 24 and 48 h) respectively. The cells were then stained with PI and analyzed DNA content by flow cytometry. After exposure to 25 μg/ml of the TGA, there was an increase of cells in G_0_/G_1 _when compared to the DMSO solvent control and a concomitant decrease of cells in S and G_2_/M phases. After treatment with 50 and 100 μg/ml of TGA, there was a decrease of cells in G_0_/G_1 _and an increase of cells in G_2_/M. Moreover, the sub-G_1 _apoptotic peak was induced by TGA in dose- and time- dependent manners (Fig 4). This indicates that TGA contains more than one component with the more active or abundant component inducing G_0_/G_1 _arrest and the less active component inducing G_2_/M arrest and/or individual component(s) in TGA exert different effects at different concentrations.

### Upregulation of Bax/Bcl-2 ratio

**Figure 5:**
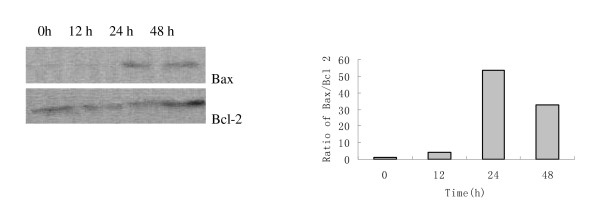
**Regulation of Bax and Bcl-2 protein expression on HepG2 cells by TGA**. Cellular lysate protein (50 μg/lane) was loaded on a 10% SDS-polyacrylamide gel, electrophoresed, and subsequently transferred onto nitrocellulose. Immunoblots were detected with antibody specific for Bcl-2 and Bax. Lysates were from HepG2 cells treated with 50 μg/ml TGA for 0, 12, 24 and 48 h, respectively. The ratio of Bax/Bcl-2 was analyzed by pImage.

The raise of the ratio of Bax/Bcl-2 is of benefit to apoptosis. In light of our study, following treatment with TGA at 50 μg/ml, pro-apoptotic protein Bax expression was up regulated in a time-dependent manner; the anti-apoptotic protein Bcl-2 was down regulated at 12 h and 24 h time points, but slightly up regulated at 48 h time point. At all events, the ratio of Bax/Bcl-2 was increased during all time points compared with control. Although the ratio of Bax/Bcl-2 at 48 h time point was decreased in some degree than 24 h time point, it was still far higher than that of control (Fig 5). The reason of this might be due to more apoptosis at 48 h in HepG2 cells negatively feedback to inhibit the ratio of Bax/Bcl-2 via enhancing the expression of anti-apoptotic protein.

### Tumor growth inhibition of implanted H_22 _cells by TGA

After tumor implantation for 24 h, administration of TGA (200, 100 or 50 mg/kg b.w., i.g) and cyclophosphamide (15 mg/kg B.W., i.p) once a day for 10 days, could significantly suppress the growth of H_22 _tumor and TGA at 200 mg/kg was more effective than the lower dosages. One-way ANOVA followed by Dunnett's test was used for statistic analysis and significant differences were found for treatment groups *vs*. control (Table 2). In addition, marked body weight loss was observed in cyclophosphamide-treated group compared to the control group, whereas only slight body weight loss was observed in TGA-treated groups. This implies that TGA might be a promising antitumor agent with low toxicity.

**Table 2:** Tumor growth inhibitory effect of TGA on H_22 _cells (mean ± SD, n = 10)

Samples	Dosage (mg/kg)	Tumor weight (g)	Growth inhibition %
Control	-	3.28 ± 1.27	
Cyclophosphamide	15	0.93 ± 0.45^a^	71.67
TGA	200	1.64 ± 0.76^b^	49.92
TGA	100	1.99 ± 0.82^c^	39.30
TGA	50	2.06 ± 1.30^c^	36.98

^a^*p *< 0.001, ^b^*P *< 0.01, ^c^*p *< 0.05 *vs *control.

## Discussion

A major complication of chemotherapy is toxicity to normal cells, which is due to the inability of drugs to differentiate between normal and malignant cells. This often impacts the efficacy of the treatment and even makes it impossible to cure the patients. One of the requisite of cancer chemopreventive agent is elimination of damaged or malignant cell through cell cycle inhibition or induction of apoptosis without or with less toxicity in normal cells [20,21].

First we investigated the cytotoxicity of TGA in HepG2 cells and primary cultured normal mouse hepatocytes. The primary cultured mouse hepatocytes were chosen as normal cells to seek selective hepatoma cytotoxic agents, because these primary cultured cells closely resemble normal cells *in vivo*. Our results indicate that TGA has relatively selective cytotoxicity to hepatoma cells based on the higher IC_50 _value in the primary cultured normal hepatocytes than that of carcinoma HepG2 cells. The relative selective cytotoxicity of TGA in HepG2 cells may be due to some of the relative selective cytotoxic components 23-, 24- and 25-O-acetylcimigenol-3-O-β-D-xylopyranoside, 25-anhydrolcimigenol-3-O-β-D-xylopyranoside and hepatoprotective constituent cimigenol xylopyranoside in it [13,22,23].

Cell proliferation is governed by the cell cycle, which is the target of many anti-cancer agents. Previous studies have demonstrated that extracts and some constituents of rhizomes of C. *racemosa*, the same genus as C. *dahurica*, possess cytotoxic activity against estrogen receptor positive (MCF-7) and estrogen receptor negative (MDA-MB231 and MDA-MB-453) human breast carcinoma cell lines by induction of cell cycle arrest and apoptosis; furthermore, glycosidic fraction could induce G_0_/G_1_cell cycle arrest when tested at 30 μg/ml and G_2_/M arrest when tested at 60 μg/ml in MCF7 cells [15,24]. In addition, it was found that actein and a fraction of black cohosh potentiated antiproliferative effects of chemotherapy agents on human breast cancer cells in more recent research [25]. In light of our study, TGA could induce G_0_/G_1 _cell cycle arrest at lower concentration (25 μg/ml) and G_2_/M arrest at higher concentration (50 and 100 μg/ml). This suggests that TGA contains more than one component with the more active or abundant component inducing G_0_/G_1 _arrest and the less active component inducing G_2_/M arrest. Active components either for G_0_/G_1 _or G_2_/M cell cycle arrests have been detected in TGA by our previous studies. 23, 24 and 25-O-acetylcimigenol-3-O-β-D-xylopyranoside, isolated from TGA could induce G_2_/M arrest [13]; while 25-anhydrolcimigenol-3-O-β-D-xylopyranoside, which exists in TGA, could induce G_0_/G_1 _arrest [26]. There might be some other potent G_0_/G_1 _active components undiscovered.

Apoptosis is a tightly regulated process, which involves changes in the expression of a distinct set of genes [27,28]. Two of the major genes responsible for regulating mitochondrial apoptosis pathway are antiapoptotic Bcl-2 and proapoptotic bax [29-31]. In particular, Bax can homodimerize with itself and heterodimerize with Bcl-2 or Bcl-xL. It appears that Bax homodimers activates apoptosis while heterodimers inhibits the process [32]. Moreover, an elevated intracellular ratio of Bax to Bcl-2 occurs during increased apoptotic cell death [33]. In our study, pronounced apoptotic cells were found in HepG2 cells treated with TGA by fluorescence staining and flow cytometric analysis. Moreover, further study showed that enhanced ratio of Bax/Bcl2 at all time points contributed to TGA induced apoptosis. The attenuation of ratio of Bax/Bcl-2 at 48 h time point than that of 24 h might be the way of self-protection for cell survival. More apoptosis at 48 h might in turn, attenuate the increased ratio of Bax/Bcl-2 by negative feedback.

## Conclusion

In conclusion, for the first time, the potential anticancer activity and the underlying mechanisms of TGA against hepatoma were investigated in this study. TGA exhibited relative cytotoxicity to HepG2 cells *in vitro *and inhibited growth of H_22 _tumor *in vivo*. The results of this study suggest that TGA might be a promising anti-hepatoma agent. Apoptosis and cell cycle arrest could be attributed, in part to its proliferating inhibition, and alteration of ratio of Bax/Bcl-2 might be one of possible mechanisms of TGA inducing apoptosis.

## Competing interests

The author(s) declare that they have no competing interests.

## Authors' contributions

ZT was mainly responsible for the experimental design, the bioactivity assays and the manuscript writing; JS and LZ did all the phytochemical work; QC did triterpene content determination and wrote the draft of that part; SC, PX and EW participated in the experimental design, technique and financial support, and manuscript writing. All authors have contributed to this work, read and approved the final manuscript.

## Pre-publication history

The pre-publication history for this paper can be accessed here:


